# Expanding equity measurements of family planning beyond wealth status and contraceptive use

**DOI:** 10.2471/BLT.20.279604

**Published:** 2021-08-31

**Authors:** Sara Stratton, Karen Hardee, Erika Houghtaling, Shawn Malarcher, Ian Askew, Maria Carrasco, Venkatraman Chandra-Mouli, Rodolfo Gomez Ponce de Leon, Jennie Greaney, Baker Maggwa, Donna R McCarraher, Jill M Peterson, Laura Raney

**Affiliations:** a Palladium, Health Policy Plus Project, 1331 Pennsylvania Ave, NW, Suite 600, Washington, DC, 20004, United States of America (USA).; b What Works Association, Arlington, USA.; c Office of Population and Reproductive Health, United States Agency for International Development, Washington, DC, USA.; d Department of Sexual Reproductive Health and Research, World Health Organization, Geneva, Switzerland.; e Latin American Center for Perinatology, Women and Reproductive Health, Pan American Health Organization, Washington, DC, USA.; f United Nations Population Fund, Commodity Security Branch, New York, USA.; g FHI 360, Results 4 Success, Durham, North Carolina, USA.; h FP2030, North America and Europe Hub, Washington, DC, USA.

Despite progress, the vision of the International Conference on Population and Development to achieve universal access to sexual and reproductive health services, including contraception, remains unfulfilled. *Transforming our world: the 2030 agenda for sustainable development* acknowledges the continuing need for sexual reproductive health and contraception by including two goals with targets aimed at universal access to contraceptive services. Realizing these goals will require greater focus and investment, to understand and address the barriers that millions of women and girls currently at risk of an unwanted pregnancy face in accessing and using voluntary family planning.

The partnership on High Impact Practices in Family Planning synthesizes and translates evidence and learning in family planning programmes to assist stakeholders in making evidence-informed decisions to maximize the impact of resources and extend voluntary, high-quality services to everyone. A persistent challenge in realizing this vision is reaching those not well served by current programmes. To communicate the evidence base to better support countries in addressing this challenge, the partnership reviewed existing definitions, frameworks and evidence from intervention studies, and secondary analyses of national surveys, policy papers and grey literature relevant to equity in family planning.^1^ The review identified eight single-intervention studies on overcoming inequities conducted in low- and middle-income countries (Afghanistan, Burundi, Cambodia, Kenya, Pakistan, Rwanda and United Republic of Tanzania) between the years 2000 and 2018, as well as many analyses of national surveys. The eight studies, which showed mixed results, revealed variations in how equity is defined, with most addressing economic barriers to contraceptive use.^1^ Secondary analysis of national surveys analysed across time showed more consistent reductions in the equity gap for key family planning outcomes.^1^ While this type of analysis implies these programmes have been successful in expanding access to key populations, it does not elucidate how this success was achieved.

Drawing on these findings, we propose a more comprehensive approach for examining and addressing inequities in family planning. We aim to challenge researchers and advocates to expand their vision of equity towards a more inclusive and insightful analysis; to encourage managers and evaluators to incorporate a more nuanced approach in defining and evaluating success; and to support implementers in thinking more creatively about the root causes of inequity and programme responses, rather than to set out indicators or provide programmatic guidance in their use. We recommend expanding how inequities are characterized and how they are measured and evaluated to go beyond wealth as the sole driver of inequity and contraceptive use as the primary outcome.

## Moving beyond wealth

How equity is defined is critical to determining where inequities exist, and often shapes programme response. The World Health Organization (WHO) defines equity in health as “the absence of avoidable, unfair, or remediable differences among groups of people, whether those groups are defined socially, economically, demographically or geographically or by other means of stratification.”^2^ In relation to family planning, FP2030 ‒ a global partnership to empower women and girls through rights-based family planning ‒ adds that “quality, accessibility and availability of contraceptive information and services should not vary by non-medically indicated characteristics, such as age, geographical location, language, ethnicity, disability, HIV [human immunodeficiency virus] status, sexual orientation, wealth, marital or other status.”^3^ These definitions go beyond wealth as the standard independent variable proxy for inequity and encourage examination of other socially determined differences that can affect equity. In addition, these definitions focus on the availability, accessibility, acceptability and quality of health care.

Evidence suggests that economics may not be the most important driver of all inequities, nor is it necessarily co-linear with other dimensions of inequity, an insight critical to designing effective approaches to addressing access issues. For example, being an adolescent or unmarried can be a limiting factor for accessing contraception, regardless of wealth. Among the youth population in 33 countries in Africa, Asia and Latin America and the Caribbean, education and marital status are as predictive of ability to use contraception as wealth status.^4^ While residence (urban versus rural) is commonly considered an indication of status, proximity to services may be a better predictor of equity in access. One study found that individuals who lived closer to health facilities had higher rates of contraceptive use compared to those living further away, regardless of their residential status.^5^ Studies such as these reinforce the need for more comprehensive and nuanced analyses and understanding of how social determinants influence health for entire population groups.

Frameworks that support operationalizing the availability, accessibility, acceptability and quality of health care will accelerate the shift to programming that addresses all dimensions of inequity. Building on social gradients of health,^6^ WHO’s Priority Public Health Conditions Analytical Framework is useful for defining the underlying determinants of health.^7^ The framework is intended to identify social determinants at play and their contribution to inequity, for example pathways, magnitude and social gradients; promising entry points for intervention; potential adverse side-effects of eventual change; possible sources of resistance to change; and what has been tried and what were the lessons learnt.^5^

Using this framework, researchers found that the burden of unintended pregnancies is not equally distributed within countries and that social determinants hinder access to contraceptives.^8^ The researchers showed that access to the health system plays a large role in helping women avoid unintended pregnancies; however, other vulnerabilities related to user characteristics, namely being from a rural area, an adolescent or a migrant, having little or no education, lacking autonomy or having been exposed to sexual violence or child marriage, hindered access. They concluded with several macro- and microlevel interventions to improve accessibility, availability and acceptability of services.^8^ This analysis shows that the WHO framework is useful to analyse the public health conditions at the differential health outcomes level to determine the root cause of differences between groups, design an intervention at each promising entry point, and ensure rigorous measurement.^8^ More work is needed to translate this and other equity frameworks into practical programming guidance and tools.

## Outcome measures

Adopting a consistent, relevant and actionable approach to measuring equity in family planning is key to identifying population groups that face access barriers. Doing so would also facilitate identifying aspects of such services that are inequitable and effective strategies for monitoring progress towards reducing inequity. The current focus on contraceptive use for measuring inequity is insufficient as it lacks dimensions of variations in fertility preferences, access and individual choice.

Equitable access to family planning services does not mean that all groups use contraception at equal rates. Rather, equity implies that all groups have the same access to information and quality services, including access to all available methods of contraception, their removal and high-quality care that includes equal treatment by providers. Furthermore, equity implies that women and men, including adolescents and young adults, can make decisions about their fertility and their use of contraceptives, and act on those decisions. Uniformly high contraception use is equitable only if it adheres to the choices of individuals in all groups.

Programmes and research should incorporate measures of equity that go beyond contraceptive use and track both demand- and supply-side barriers to access to family planning services, methods, information and quality as measured by client satisfaction, respectful care and equitable treatment.^9^ Outcome measures could include demand satisfied for contraception, unintended or mistimed pregnancy or childbearing, ability to achieve preferred number of children and rights-reflective indicators such as agency to make decisions, including about voluntary contraceptive use,^10^ and interruptions in the availability of contraception, for example in crisis situations.

## Moving forward

A growing number of tools and resources to help planners and advocates adopt a more comprehensive approach to equity exist. The *Equity in family planning strategic planning guide* published by the High Impact Practices partnership leads readers through steps to work towards equity in family planning.^11^ The guide encourages the identification of those groups whose needs are not being met as well as the barriers they face, and suggests tools to meet these needs. The *Approach for diagnosing inequity in family planning programs: methodology and replication guide*^12^ uses demographic and health surveys to identify inequities across the dimensions of availability, accessibility, acceptability and quality of health care and in demand satisfied, based on age, education, marital status, wealth, residence, religion and ethnic status. When sufficient data are available, this tool can also be used to analyse inequities down to the subnational level.

The FP2030 Performance Monitoring and Evidence Working Group and the United States Agency for International Development-supported Equity in Family Planning taskforce are two initiatives that are working to identify and define key indicators for monitoring and evaluating progress in equity.

The implications for this expanded view of programming to address equity may mean investing in interventions and studies outside of and in addition to family planning health care, such as those relating to shifting gender norms, education and child survival, as well as collecting sufficient data to be able to disaggregate or control for relevant different dimensions of inequity.

Adopting these recommendations can contribute to addressing underlying constraints to individuals and couples’ access to and demand for contraceptives based on inequities and will help reach the millions of women and girls in need of quality reproductive health care.
